# Pitaya HpWRKY3 Is Associated with Fruit Sugar Accumulation by Transcriptionally Modulating Sucrose Metabolic Genes *HpINV2* and *HpSuSy1*

**DOI:** 10.3390/ijms20081890

**Published:** 2019-04-17

**Authors:** Wei Wei, Mei-nv Cheng, Liang-jie Ba, Run-xi Zeng, Dong-lan Luo, Yong-hua Qin, Zong-li Liu, Jian-fei Kuang, Wang-jin Lu, Jian-ye Chen, Xin-guo Su, Wei Shan

**Affiliations:** 1 State Key Laboratory for Conservation and Utilization of Subtropical Agro-bioresources/Guangdong Provincial Key Laboratory of Postharvest Science of Fruits and Vegetables/Key Laboratory of Biology and Genetic Improvement of Horticultural Crops (South China) of Ministry of Agriculture/Engineering Research Center of Southern Horticultural Products Preservation, Ministry of Education, College of Horticulture, South China Agricultural University, Guangzhou 510642, China; weiwei_11663@163.com (W.W.); cc13424454013@126.com (M.-n.C.); zengrunxi@126.com (R.-x.Z.); qinyh@scau.edu.cn (Y.-h.Q.); liuzongli@scau.edu.cn (Z.-l.L.); jfkuang@scau.edu.cn (J.-f.K.); wjlu@scau.edu.cn (W.-j.L.); chenjianye@scau.edu.cn (J.-y.C.); 2 School of Food and Pharmaceutical Engineering, Guizhou Engineering Research Center for Fruit Processing, Guiyang University, Guiyang 550003, China; baliangjie@163.com (L.-j.B.); luodonglan1991@163.com (D.-l.L.); 3 Department of Food Science, Guangdong Food and Drug Vocational College, Guangzhou 510520, China

**Keywords:** fruit maturation, fruit quality, sucrose-hydrolyzing enzyme genes, transcriptional activation

## Abstract

Sugar level is an important determinant of fruit taste and consumer preferences. However, upstream regulators that control sugar accumulation during fruit maturation are poorly understood. In the present work, we found that glucose is the main sugar in mature pitaya (*Hylocereus*) fruit, followed by fructose and sucrose. Expression levels of two sucrose-hydrolyzing enzyme genes *HpINV2* and *HpSuSy1* obviously increased during fruit maturation, which were correlated well with the elevated accumulation of glucose and fructose. A WRKY transcription factor HpWRKY3 was further identified as the putative binding protein of the *HpINV2* and *HpSuSy1* promoters by yeast one-hybrid and gel mobility shift assays. HpWRKY3 was localized exclusively in the nucleus and possessed trans-activation ability. *HpWRKY3* exhibited the similar expression pattern with *HpINV2* and *HpSuSy1*. Finally, transient expression assays in tobacco leaves showed that HpWRKY3 activated the expressions of *HpINV2* and *HpSuSy1*. Taken together, we propose that HpWRKY3 is associated with pitaya fruit sugar accumulation by activating the transcriptions of sucrose metabolic genes. Our findings thus shed light on the transcriptional mechanism that regulates the sugar accumulation during pitaya fruit quality formation.

## 1. Introduction

In recent years, following the development of the economy and the improvement of people’s living standard, as well as the people’s multiple demands, special or rare fruit varieties in the markets are highly favored by consumers. Pitaya (*Hylocereus*) fruit, also called dragon fruit, is generating considerable consumer attention in many tropical and subtropical countries with exotic appearance, striking colors, high nutrients, and a delicious taste [1,2]. For example, pitaya juicy flesh exhibits different colors varying from white, yellow, pink, orange, red, or purple [3]. More attractively, pitaya fruits are rich in bioactive compounds such as polyphenol, flavonoid, and betalains, which is evidenced by lots of studies that focus on metabolite profiling and analysis of antioxidant and antiproliferative activities in ripe fruits [1,3,4,5,6]. Recently, structural genes involved in pitaya betalain biosynthesis including *TYR*, *DOD-like*, *CytP450-like,* and *GT-like*, were identified using RNA sequencing [7], and the gene *CytP450-like*, was further found to be controlled by a regulatory protein WRKY transcription factor (TF) HpWRKY44 [8]. Fruits also accumulate different types of soluble sugars during maturation. Soluble sugars composition (sucrose, glucose, and fructose) and concentration at harvest has been proposed to be an important contributor to fruit flavor and taste/sweetness, thus impacting consumers’ preferences [9,10]. However, much remains elusive about sugar accumulation during pitaya fruit quality formation. 

In most higher plants, soluble sugars are synthesized in sinks. This is achieved by the activities of various enzymes and/or transcription levels of genes associated with sugar metabolic and transport processes [9,10,11]. Generally, the breakdown of sucrose into glucose, fructose, or UDP-glucose is mainly mediated by three enzyme families, invertases (INVs), sucrose synthases (SuSys), and sucrose phosphate synthases (SPSs) [11]. In fruits like peach, apple, and watermelon [12,13,14,15], genes encoding INVs, SuSys, and SPSs have been identified and their expressions are closely related to sugar accumulation during fruit development and ripening. Interestingly, it has been found recently that the ectopic overexpression of an Arabidopsis MYB TF *AtMYB12* leads to a significant reduction in soluble sugars levels in tomatoes [16], while rice plants overexpressed a NAC TF *NAC066* accumulate higher contents of soluble sugars [17]. Nevertheless, how and by what means TFs modulate soluble sugars accumulation during fruit maturation are largely unknown. 

As a superfamily of plant-specific TFs, WRKY proteins have been extensively studied in a wide range of plants, and implicated to play roles in diverse biological processes, including plant growth and development, senescence, biotic and abiotic stress responses, and phytohormones signaling [18,19,20,21]. Notably, accumulating evidence demonstrate that WRKY TFs also participate in regulating the production of valuable plant metabolites such as phenylpropanoids, alkaloids, and terpenes [22,23]. For instance, *Withania somnifera* WsWRKY1 positively modulates triterpenoid withanolides production by the direct regulation of genes involved in triterpenoid pathway [24]. CsWRKY31 and CsWRKY48 negatively regulate O-methylated catechin biosynthesis in tea plants through repressing *CsLAR*, *CsDFR*, and *CCoAOMT* that associate with *O*-methylated catechin biosynthesis [25]. In grapevines, VvWRKY03 and VvWRKY24 are found to activate stilbene synthase gene *VvSTS29*, thereby acting as positive regulators of stilbene biosynthesis [26], while VvWRKY8 negatively controls stilbene biosynthesis by the repression of *VvSTS15/21* expression [27]. These reports highlight the regulatory roles of WRKY TFs in plant metabolite biosynthesis. In the case of soluble sugars, the regulatory mechanisms of WRKY TFs involved in modulating sugar metabolism, especially during fruits quality formation, remain to be elucidated. 

The objective of this work was to investigate how WRKY TFs affect soluble sugars accumulation during pitaya fruit maturation. A pitaya fruit WRKY TF HpWRKY3 was identified and characterized, and it was shown that HpWRKY3 is associated with fruit sugar accumulation by the activation of sucrose metabolic genes *HpINV2* and *HpSuSy1*.

## 2. Results and Discussion

### 2.1. Changes in Soluble Sugars during Pitaya Fruit Maturation

**Figure 1:**
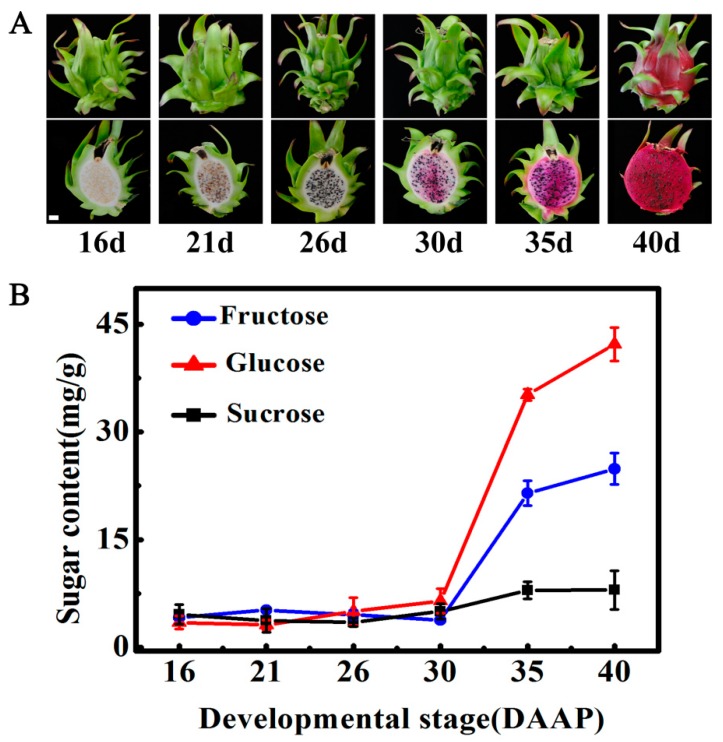
Glucose is the main sugar in mature pitaya fruit. (**A**) Photograph of pitaya fruit at different developmental stages. (**B**) Changes of soluble sugars (glucose, fructose, and sucrose) contents during fruit maturation. Fruit at 16, 21, 26, 30, 35, and 40 days after artificial pollination (DAAP) was sampled for analysis. Data represent mean values from three biological replicates (± S.E.).

For pitaya fruit with red skin and pulp, pulp color starts to turn red with the accumulation of betalains, which is an indicator of the initiation of fruit maturation [2,7,8]. As shown in Figure 1A, pulp color began to turn red at 30 DAAP and developed fully red at 40 DAAP. 

Several reports have shown that glucose and fructose are the main soluble sugars in mature pitaya fruit, and they obviously accumulate during maturation [2,28,29]. Consistent with these previous results, levels of glucose and fructose in pitaya pulp were significantly high at maturity, being 42.2 mg/g and 24.9 mg/g at 40 DAAP, respectively, while the concentration of sucrose was 8.0 mg/g (Figure 1B). In addition, both glucose and fructose rapidly accumulated following maturation initiated at 30 DAAP, and their levels increased gradually afterwards. At 40 DAAP, the contents of glucose and fructose were ~12.2- and ~6.0-fold of the levels at 16 DAAP, respectively (Figure 1B). The value of sucrose content remained almost constant during maturation (Figure 1B).

### 2.2. Expression Levels of HpINV2 and HpSuSy1 Were Positively Correlated with Accumulations of Glucose and Fructose

**Figure 2:**
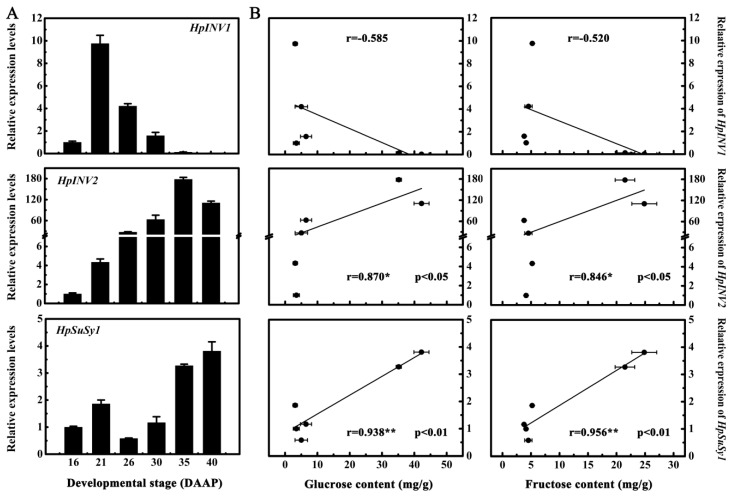
The expression of *HpINV2* and *HpSuSy1* are correlated with glucose and fructose accumulation during pitaya fruit maturation. (**A**) The expression patterns of sucrose metabolic genes *HpINVs* and *HpSuSy1* in pitaya fruit at different developmental stages. Fruit at 16, 21, 26, 30, 35, and 40 days after artificial pollination (DAAP) was sampled for analysis. Expression values are means ± S.E. of three biological replicates normalized using *ACTIN* as an internal control. (**B**) Correlation between *HpINVs* and *HpSuSy1* expression levels and contents of glucose and fructose. * and ** represent significant correlation at 0.05 and 0.01 level respectively determined by SPSS Statistics 20.0.

Sucrose is well-documented to be irreversibly hydrolyzed into glucose and fructose by INVs and SuSys [11]. Research on tomatoes [30,31], peach [12,13], pineapple [32], and watermelon [15] have demonstrated that both INVs and SuSys are encoded by multi-gene members. Moreover, enzyme activities and/or gene expression levels of INVs and SuSys are found to be pivotal for soluble sugars accumulations during fruit development and maturation [11,33]. Due to the lack of pitaya genome, little information is available about the sucrose metabolism during pitaya fruit maturation. From our previous transcriptome database associated with pitaya fruit quality formation during maturation [7], two genes encoding INVs termed *HpINV1* and *HpINV2*, as well as one gene encoding SuSys termed *HpSuSy1*, were annotated and found to be differentially expressed during fruit maturation. To investigate the possible relationship between expression levels of *HpINV1*, *HpINV2,* and *HpSuSy1*, and soluble sugars accumulations during pitaya fruit maturation, the transcript levels of *HpINV1*, *HpINV2,* and *HpSuSy1* were quantified by qRT-PCR. The results showed that, similar to the accumulation patterns of glucose and fructose during pitaya fruit maturation, the transcript levels of *HpINV2* and *HpSuSy1* were dramatically elevated after 30 DAAP, peaking at 35 and 40 DAAP, respectively (Figure 2A). *HpINV1* was induced at the early stage (21 DAAP), while obviously decreased following the glucose and fructose accumulations after 30 DAAP (Figure 2A). Moreover, linear regression analysis indicated that both *HpINV2* and *HpSuSy1* transcripts were positively correlated with contents of glucose and fructose (Figure 2B). On the contrary, no significant correlation was found between *HpINV1* transcripts and glucose or fructose contents (Figure 2B). Therefore, these data suggest that *HpINV2* and *HpSuSy1* are good candidates associated with sugar accumulation during pitaya fruit maturation.

### 2.3. Identification of HpWRKY3 as A Putative Transcriptional Regulator of HpINV2 and HpSuSy1

Besides structural genes, the identification of regulatory proteins like TFs that control the expression of *HpINV2* and *HpSuSy1* will provide crucial clues to elucidate the regulatory network associated with sugar accumulation during pitaya fruit maturation. Then Y1H screening was carried out to uncover the TFs that target *HpINV2* and *HpSuSy1*. Using the promoter of *HpINV2* and *HpSuSy1* as bait, one WRKY TF was successfully identified. Since the WRKY shares high similarity of 75% and 57% to sugar beet BvWRKY3 and Arabidopsis AtWRKY3 within the NCBI database, it was designated as HpWRKY3. 

**Figure 3:**
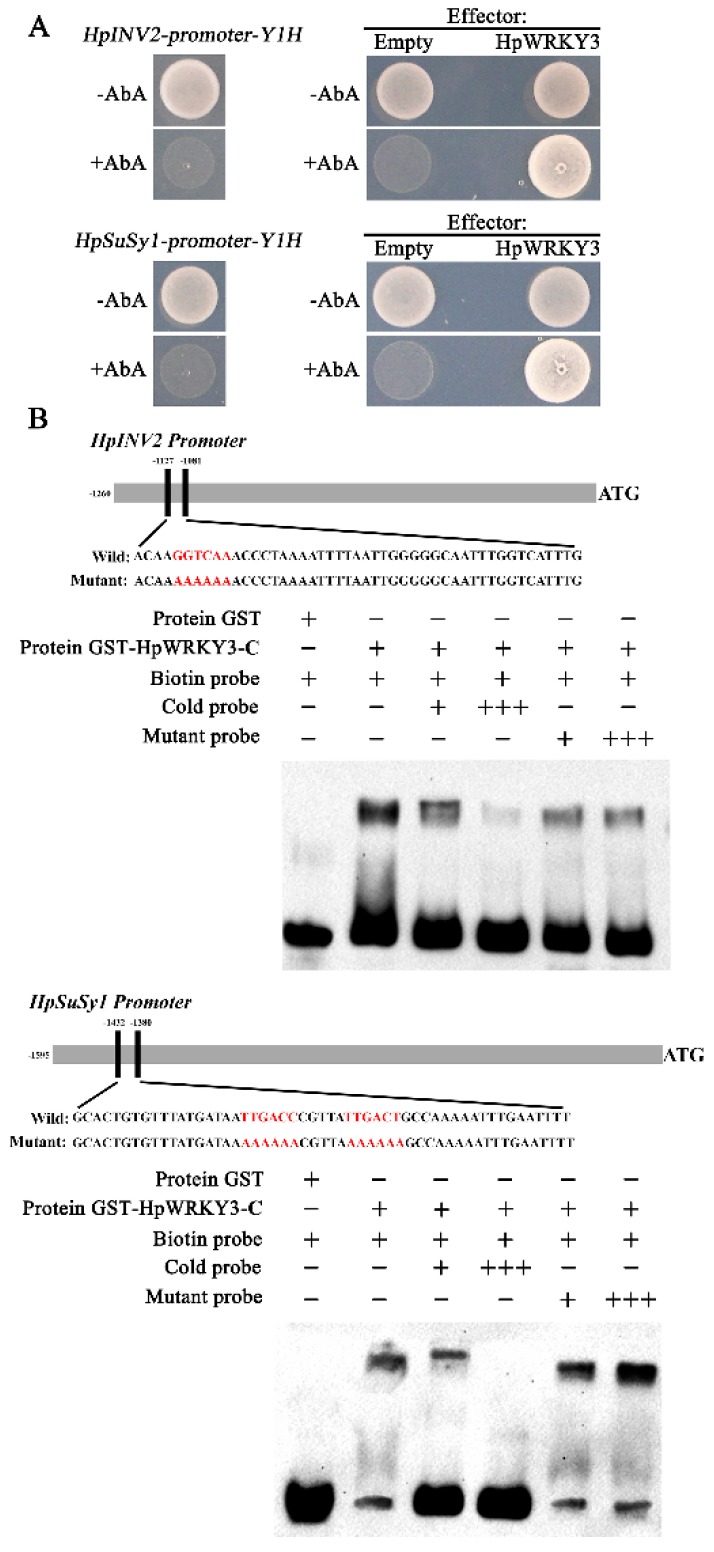
HpWRKY3 directly bind to *HpINV2* or *HpSuSy1* promoter. (**A**) Y1H assay showing the physical interaction of the HpWRKY3 protein with *HpINV2* or *HpSuSy1* promoter. (**Left**) No basal expression of *HpINV2*- or *HpSuSy1*-pro was detected in yeast grown on synthetic dropout (SD) medium lacking Leu (SD/−Leu) in the presence of 500 ng/mL Aureobasidin A (AbA) for stringent selection. (**Right**) Yeast growth assays were performed after the Y1H reporter strains were transformed with plasmids carrying cassettes constitutively expressing HpWRKY3 effector or empty (pGADT7, negative control). Interaction was determined based on the ability of transformed yeast to grow on SD/−Leu in the presence of AbA. All suspensions of yeast cells in this assay were adjusted to OD_600_ = 0.1. (**B**) Electrophoretic mobility shift assay (EMSA) showing the binding of HpWRKY3 to the W-box of the *HpINV2* or *HpSuSy1* promoter. Purified glutathione-S-transferases (GST)-tagged HpWRKY3 protein were incubated with the biotin-labeled wild-type probe containing W-box, and the DNA–protein complexes were separated on native polyacrylamide gels. Sequences of both the wild-type and mutated probes are shown at the top of the image (wild-type and mutated W-box are marked with red letters). The probe with the mutated W-box was used to test binding specificity. Shifted bands, suggesting the formation of DNA–protein complexes, are indicated by arrows. ‘‘−’’ represents absence, ‘‘+’’ represents presence. ‘‘+++’’ indicates increasing amounts of unlabeled or mutated probes for competition experiments.

The interactions between HpWRKY3 and promoters of *HpINV2*/*HpSuSy1* were further confirmed through Y1H assay. As shown in Figure 3A, in the presence of Aureobasidin A (AbA), the basal activity of *HpINV2* and *HpSuSy1* promoter was not detected in yeast. However, yeast cells co-transformed with HpWRKY3 and *HpINV2* or *HpSuSy1* could grow on the medium containing AbA, which suggests that HpWRKY3 binds to *HpINV2* or *HpSuSy1* promoter to induce the expression of the resistance gene *AbA* driven by the *HpINV2* or *HpSuSy1* promoter (Figure 3A). 

Previous studies on the recognition sites of WRKY TFs have revealed that they preferentially bind to the W-box [(C/T)TGAC(C/T)] motif in the promoter sequences of their downstream targets [18,34]. An in vitro EMSA was also conducted to verify the binding of HpWRKY3 to *HpINV2* and *HpSuSy1* promoters. DNA fragments containing W-box motif derived from *HpINV2* and *HpSuSy1* promoters (Text S1, Tables S1 and S2) were biotin-labelled and incubated with purified recombinant HpWRKY3-C protein (Figure S1), causing clear mobility shifts in bands (Figure 3B). Addition of the excess unlabeled probes for competition abolished the bands in a dosage-dependent manner, but the mutant probes did not affect the binding (Figure 3B). The results of EMSA and Y1H assay collectively demonstrate that HpWRKY3 may act as a putative transcriptional regulator of *HpINV2* and *HpSuSy1* by binding to their promoters via the W-box elements. 

### 2.4. Bioinformatics and Molecular Characteristics of HpWRKY3

**Figure 4:**
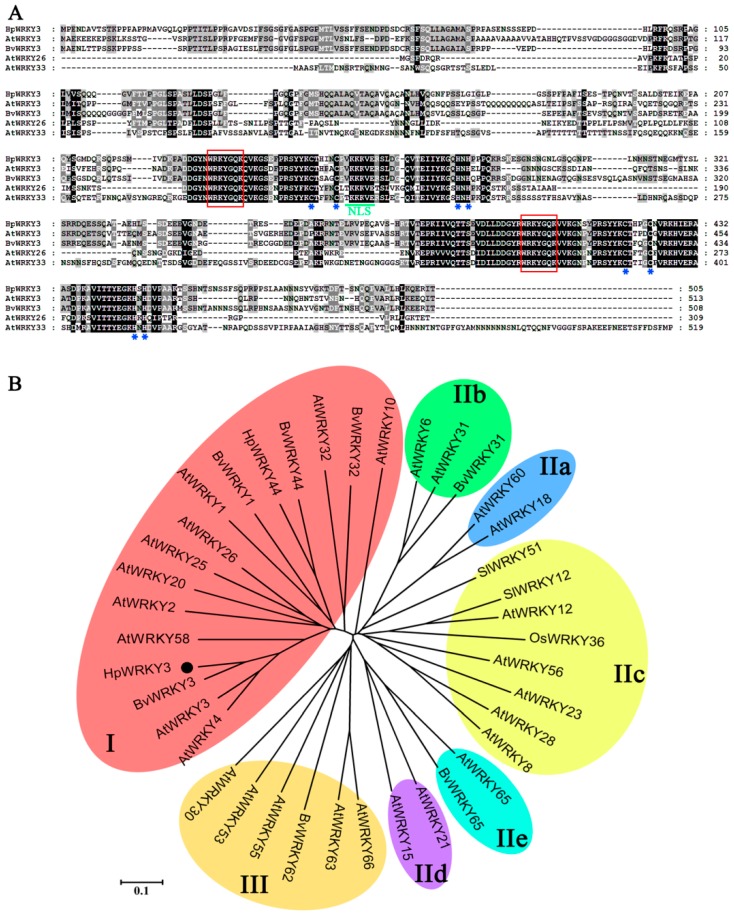
Multiple sequence alignment and phylogenetic analysis of HpWRKY3. (**A**) Multiple alignment of HpWRKY3 with other WRKY members. The following proteins were used for analysis: AtWRKY3 (NP_178433.1), BvWRKY3 (XP_010683088.1), AtWRKY26 (NP_196327.1), and AtWRKY33 (NP_181381.2). Identical and similar amino acids were shaded in black and grey, respectively. The WRKY domains and the zinc-finger structures are boxed and marked by asterisks, respectively. (**B**) Phylogenetic analysis of WRKYs. HpWRKY3 was highlighted with black circle. The phylogenetic tree was constructed with neighbor-joining test using MEGA program (version 5.0).

HpWRKY3 has an open reading frame (ORF) of 1515 bp length, encoding a protein with 505 amino acid residues, calculated molecular weight of 54.89 kDa, and *p*I value of 6.71 (Text S2). The most noticeable property of WRKY proteins is the presence of one or two WRKY domain in their N-terminal, which is approximately 60 amino acids residues including an invariant WRKYGQK core sequence [35]. Two types of zinc-finger motifs, like C_2_H_2_ (C–X_4–5_–C–X_22–23_–H–X_1_–H) and C_2_HC (C–X_5–7_–C–X_23_–H–X_1_–C), are usually observed at the C-terminal of WRKY proteins [36]. WRKY proteins are generally classified into three distinct groups (I–III) according to the number of WRKY domains and the type of the zinc-finger motif, among which the group II is further split into five subgroups (IIa–e) [35,36]. As shown in Figure 4A, multiple-alignment of HpWRKY3 with several reported WRKYs showed that, similar to group I WRKYs such as Arabidopsis AtWRKY3 and AtWRKY33 [35,36], HpWRKY3 contains two WRKY domains and a C_2_H_2_ zinc-finger motif. The phylogenetic tree also showed that HpWRKY3 together with AtWRKY3 and AtWRKY33, belong to group I (Figure 4B). 

**Figure 5:**
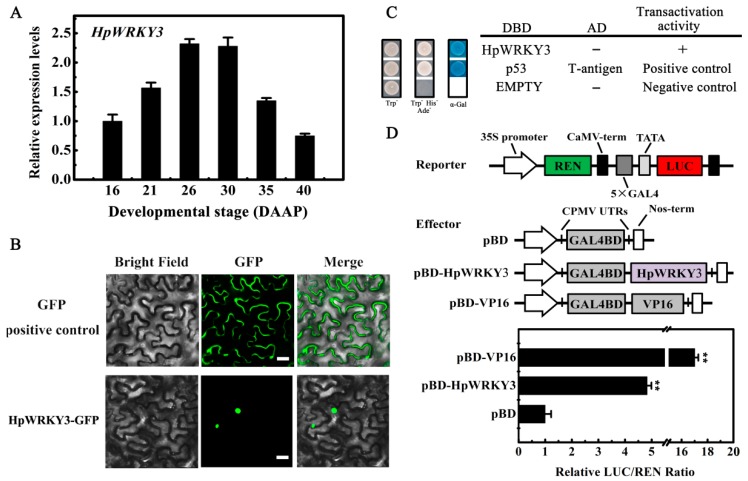
Molecular properties of HpWRKY3. (**A**) Temporal expression patterns of *HpWRKY3* during pitaya fruit maturation. Fruit at 16, 21, 26, 30, 35, and 40 days after artificial pollination (DAAP) was sampled for analysis. Expression values are means ± S.E. of three biological replicates normalized using *ACTIN* as an internal control. (**B**) Subcellular localization of HpWRKY3 in epidermal cells of tobacco leaves. A plasmid harboring GFP or HpWRKY3-GFP was transformed into *Nicotiana benthamiana* leaves by *Agrobacterium tumefaciens strain* EHA105. GFP signals was observed with a fluorescence microscope after 2 d of infiltration. Bars, 30 μm. (**C**) Transcriptional activation of HpWRKY3 in yeast cells. The coding region of HpWRKY3 was inserted into the pGBKT7 (GAL4DBD) to create the pGBKT7-HpWRKY3 construct. The yeast cells of strain Y2HGold harboring the pGBKT7-HpWRKY3 plasmids were grown on SD plates without tryptophan (Trp^−^) or without tryptophan, histidine, and adenine (Trp^−^His^−^Ade^−^) for three days at 28 °C, followed by the α-galactosidase assay (α-Gal staining). pGBKT7 and pGBKT7-53 + pGADT7-T were used as negative and positive control, respectively. (**D**) Trans-activation of HpWRKY3 in *Nicotiana benthamiana* leaves. The trans-activation ability of HpWRKY3 was demonstrated by the ratio of luciferase (LUC) to renilla luciferase (REN). The LUC/REN ratio of the empty pBD vector (negative control) was used as a calibrator (set as 1). pBD-VP16 was used as a positive control. Data are means ± S.E. of six independent biological replicates. Asterisks represents significant differences at 0.01 level by student’s *t*-test, compared to pBD.

For systematic molecular characterization, expression profile of *HpWRK3* during pitaya fruit maturation was analyzed by qRT-PCR. As shown in Figure 5A, the transcript level of *HpWRKY3* was steadily increased from 16 to 30 DAAP, reaching the peak at 30 DAAP, following the initiation of glucose and fructose accumulations. WRKY TFs are known to be localized to nuclei [8,37]. Coding sequence of *HpWRKY3* was fused to GFP and transiently expressed in tobacco leaves. Microscopic observation of tobacco leaf epidermal cells revealed that GFP alone was expressed in the cytoplasm as well as the nucleus, whereas the signal for *HpWRKY3*-GFP was exclusively detected in the nucleus (Figure 5B). Transcriptional ability is important for TFs’ function. The transcriptional activity of HpWRKY3 was assessed in yeast cells using the GAL4 DNA-binding domain-coding sequence (GAL4BD)-based system. As shown in Figure 5C, contrary to negative control cells (pGBKT7 vector), both positive control (pGBKT7-53 + pGADT7-T) and *HpWRKY3* transformed yeast cells grew well on SD/−Trp−His−Ade plates, exhibiting α-galactosidase activities. Transcriptional activation ability of HpWRKY3 was further verified in tobacco leaves using the dual-luciferase reporter system, which showed that, like the activator control VP16, co-transformation of *HpWRKY3* with the reporter apparently increased luciferase (LUC)/renilla luciferase (REN) ratio (Figure 5D). Based on these results, it is implied that HpWRKY3 is a nuclear-localized transcriptional activator that is potentially associated with pitaya fruit soluble sugar accumulation. 

### 2.5. HpWRKY3 Enhances the Transcriptions of HpINV2 and HpSuSy1

**Figure 6:**
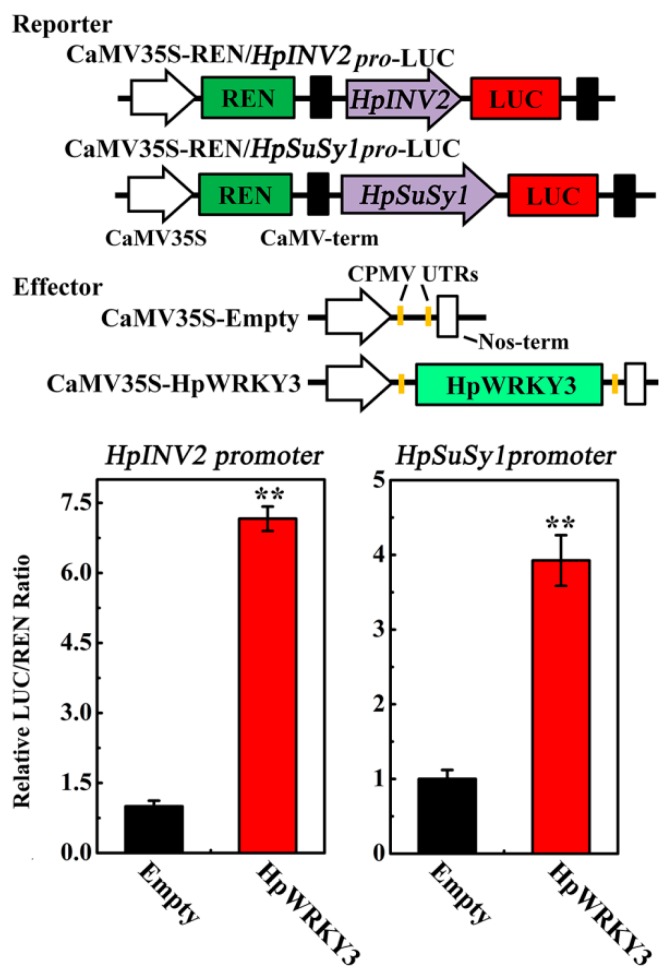
HpWRKY3 activates *HpINV2* or *HpSuSy1* transcriptions by dual-luciferase transient expression assay in *Nicotiana benthamiana* leaves. The reporter and effector vectors are illustrated in the top panel. Data are means ± S.E. of six independent biological replicates. Asterisks indicate significant differences by student’s *t*-test (** *p* < 0.01).

After figuring out *HpINV2* and *HpSuSy1* as the potential downstream targets of HpWRKY3, and establishing HpWRKY3 as a transcriptional activator, we were curious whether HpWRKY3 could directly activate the transcriptions of *HpINV2* and *HpSuSy1*. To address this particular question, we performed the transient dual-luciferase reporter assay in tobacco leaves. For this assay, *HpINV2* and *HpSuSy1* promoters were fused upstream of the *LUC* gene to serve as reporters, along with the *REN* gene driven by CaMV35S serving as an internal control. As presented in Figure 6, compared to the empty construct as the control, co-expression of HpWRKY3 with *HpINV2* and *HpSuSy1* promoters significantly elevated LUC/REN ratios, suggesting that HpWRKY3 indeed enhances the transcriptions of *HpINV2* and *HpSuSy1* in vivo. 

Besides INVs and SuSys, other sucrose metabolic enzymes including hexokinase, fructokinase, sucrose phosphate synthase, and UDP-glucose pyrophosphorylase, as well as sugar transporters, are also important factors determining fruit yield and quality by stimulating sugar accumulation. For example, transcripts of peach sucrose transporter *SUT2* and *SUT4*, and tonoplastic monosaccharide transporter *TMT2* are found to be positively correlated with fruit sucrose accumulation [12]. Induced expression levels of apple *MdTMT1*, *MdSUT2,* and *MdTMTs* have been shown to be involved in the accumulation of fruit soluble sugars [9,10]. A noticeable increase in sucrose accumulation is found in *CitSUT1*-overexpressed transgenic citrus lines [38]. Intriguingly, an apple ABA-responsive TF MdAREB2 and a watermelon sugar-induced WRKY TF SUSIWM1 were recently reported to play a crucial role in fruit sugar accumulation by the direct modulation of sugar transporter genes [9,39]. Thus, it is worthwhile to identify other sucrose metabolic and sugar transporter genes in pitaya fruit and elucidate their possible involvement in sugar accumulation, as well as to investigate whether their expressions are directly regulated by HpWRKY3. Accumulating evidence suggest that regulatory activities of WRKYs on their target genes are strengthened or weakened by other TFs and interaction proteins [40]. For example in banana fruit, it has been observed that a NAC TF MaNAC5 interacted with MaWRKY1 and MaWRKY2, activating a set of *PR* genes in the disease response [41], while the interaction of MaWRKY26 with a VQ motif-containing protein MaVQ5, suppressed the activation of JA biosynthetic genes by MaWRKY26 in response to cold stress [42]. A recent study in strawberry revealed that FaMYB44.2, FaMYB10, and FabHLH3 formed an intricate network to regulate *FaSPS3* expression during sucrose accumulation [43]. Therefore, screening and identification of HpWRKY3-interacting proteins will provide important information on the complex regulatory network of sugar accumulation during fruit quality formation.

## 3. Materials and Methods

### 3.1. Fruit Samples

A pitaya cultivar (*Hylocereus polyrhizus* cv. Guanhuahong) with red peel and pulp was selected for this work. Plants were grown in Jinsuinong commercial orchard (Madong village, Baiyun district, Guangzhou City, Guangdong Province, China) received standard horticultural practices, and disease and insect control. Based on the change of pulp color, fruits were harvested at six important stages (16, 21, 26, 30, 35, and 40 days after artificial pollination (DAAP)) from October to December 2017. At each sample point, fruits were cut into two portions for photograph. Pulps from three fruits from three different plants were sliced and ground into powder in liquid nitrogen. Thereafter, all samples were stored at −80 °C until future physiological and molecular assays. 

### 3.2. Soluble Sugar Quantification by HPLC

The extraction of sugars (fructose, glucose, and sucrose) was performed as previously described [44]. One gram of frozen pulp sample was ground to a powder in liquid nitrogen, and homogenized in 5.0 mL of ethanol (80%) at 35  °C for 20 min followed by centrifugation at 10,000 × *g* for 10 min, at 20  °C. The residue was extracted twice, and the supernatant was collected and supplemented with 80% ethanol to 25 mL. Then, 1 mL extracted solution was dried under vacuum condition (Eppendorf Concentrate Plus, Hamburg, Germany) at 45  °C, and the residue was dissolved in 0.5 mL distilled water and filtered with water syringe filter. The filtered solution of 20 μL was used for sugars analysis by the high-performance liquid chromatography (HPLC) (Agilent 1200, Agilent Technologies, Santa Clara, CA, USA), and quantity of individual sugars was calculated using peak areas of standards, as described previously [44].

### 3.3. RNA Preparation, Gene Isolation, and Bioinformatic Analysis

Total mRNA was extracted from pitaya pulp using Quick RNA Isolation Kit (Huayueyang, Beijing, China). cDNAs were synthesized from 1 μg of total RNA using the reverse transcriptase M-MLV (TaKaRa, shiga, Japan) according to the supplier’s instructions. The full-length of *HpWRKY3* gene was isolated from our transcriptome database reported previously [7]. Theoretical isoelectric points (*pI*) and mass values were assessed on the website (http://web.expasy.org/compute_pi/). WRKYs were aligned by CLUSTALW (version 1.83) and viewed using GeneDoc software. The neighbor-joining phylogenetic tree of WRKYs was obtained using MEGA program (version 5.0). 

### 3.4. Analysis of Gene Expression by qRT-PCR

cDNAs from each sample were subjected to quantitative real-time PCR (qRT-PCR) assays. qRT-PCR was performed using the step one plus a CFX96 Touch™ real-time PCR detection system (Bio-Rad, Hercules, CA, USA) and GoTaq qPCR master mix kit (Promega, Madison, WI, USA). The expression levels of target genes were normalized by using housekeeping gene *Actin* [8]. 

### 3.5. Gene Promoter Analysis

Gene promoters of *HpINV2* and *HpSuSy1* were isolated from pitaya genomic DNA by nested PCR. *Cis*-elements present in the promoters were analyzed through Plant-CARE online platform (http://bioinformatics.psb.ugent.be/webtools/plantcare/html/). 

### 3.6. Yeast One-Hybrid Assay

Yeast one-hybrid assay was performed by using Matchmaker Gold Yeast One-Hybrid System (Clontech, Mountain View, CA, USA), as described previously [45]. About 600 bp promoter of *HpINV2* and *HpSuSy1* was inserted into the reporter plasmid pAbAi and transformed into yeast strain Y1H Gold. To construct the cassette expressing the effector, the coding sequence of *HpWRKY3* was cloned into pGADT7. The resulting vector was then introduced into reporter strains. The transformed reporter strains were grown on SD/−Leu media containing 500 ng/mL Aureobasidin A (AbA) for 3 days at 28 °C to test the possible interaction. 

### 3.7. Recombinant Protein Induction, Purification, and EMSA Assay

The *E. coli* strain Transetta (DE3) was transformed with GST-HpWRKY3-C, and 5 mL of overnight-grown transformed cells were used to inoculate 500 mL of LB medium, which was incubated with shaking at 37 °C. Reaching an OD_600_ nm of 0.4–0.6, 0.3 mM isopropyl-β-*D* -thiogalactopyranoside (IPTG) was added to the cells to induce the expression of the HpWRKY3 protein for 3 h at 37 °C. After the incubation period, the cells were centrifuged and disrupted by sonication. Equilibrating with lysis buffer, the induced proteins were then affinity-purified using a Glutathione-Superflow Resin (Clontech) according to the manufacturer’s protocols, following SDS-PAGE and Coomassie Brilliant Blue staining to confirm protein size and purity.

Electrophoretic mobility shift assay (EMSA) was performed as described previously [8,46]. The synthetic nucleotides (~60 bp) containing the W-box of the 5′ UTR of *HpINV2* and *HpSuSy1* oligonucleotides were biotin-labeled at the 5′ end. The purified recombinant HpWRKY3 was incubated with biotin-labeled probes in binding buffer for 25 min at 30 °C. Competitions were carried out by adding cold probes with unlabeled DNA fragments with the same or mutant sequences. The reaction mixtures were separated on 6% native polyacrylamide gels. After electrophoresis, the DNA-protein complexes were transferred onto a nylon membrane. The signals from the labeled DNA were detected by using the LightShift chemiluminescent EMSA kit (Thermo Scientific, Rockford, IL, USA) in a ChemiDoc™ MP Imaging System (Bio-Rad).

### 3.8. Subcellular Localization Assay

To investigate the subcellular localization of HpWRKY3, its coding sequence was cloned into the binary vector pEAQ-GFP. *Agrobacterium tumefaciens* strain EHA105 strains harboring HpWRKY3-GFP constructs were infiltrated into tobacco (*Nicotiana benthamiana*) leaves by using a 1-mL disposable syringe as described previously [8]. After 48 h, the GFP fluorescent signal in the epidermal cells of the infiltrated leaves was detected by using a Zeiss fluorescence microscope. 

### 3.9. Trans-Activation Activity Assay in Yeast

The coding sequence of *HpWRKY3* was cloned into the vector pGBKT7. The resulting constructs were then transformed into the yeast strain Y2HGold. The MATCHMAKER GAL4-based Two-Hybrid system 3 (Clontech) was used for the transactivation activity assay according to the manufacturer’s protocol. Each yeast liquid culture was diluted serially to OD_600_ = 0.6, and 5 μL of each dilution was inoculated onto various synthetic dropout (SD) medium, and the trans-activation activity was indicated by the yeast growth and α-galactosidase activity, as described previously [47].

### 3.10. Transient Trans-Activation Assay in Tobacco Leaves

Assays were carried out as described previously [48]. The GAL4 plasmid with *firefly luciferase* (*LUC*) gene was used as a reporter, and the *renilla luciferase* (*REN*) gene in the same plasmid was used as an internal control. To determine the transcriptional regulation activity of HpWRKY3 in plant cells, the CDS of HpWRKY3 was inserted into pBD to construct pBD-HpWRKY3 as effector. The positive control (pBD-VP16) was constructed by fusing VP16, a herpes simplex virus-encoded transcriptional activator, to pBD. pBD itself was used as a negative control. To determine how HpWRKY3 activate *HpINV2* and *HpSuSy1* expression, the CDS of HpWRKY3 was introduced into pEAQ vector to generate the effector. *HpSuSy1* and *HpINV2* promoters were inserted into the dual luciferase vector pGreenII 0800 as reporters. The reporter and effector constructs were co-infiltrated into tobacco leaves mentioned above. After 48 h of infiltration, luciferase assays were performed using dual-luciferase reporter assay kit (Promega) and quantified by a Luminoskan Ascent Microplate Luminometer (Thermo Fisher Scientific, Rockford, IL, USA). The trans-activation ability of HpWRKY3 was defined by the ratio of LUC/REN. 

### 3.11. Statistics

All assays in this study were performed as at least three independent biological replicates. Values are means ± SE of three or six biological repeats. The Student’s *t*-test was applying for statistical analysis (* *p* < 0.05, ** *p* < 0.01). Correlation analysis between transcripts of sucrose metabolic genes and amounts of soluble sugars was performed by SPSS Statistics 22.0. Linear regressive analysis and scatter plots were prepared with Origin8.0.

### 3.12. Primers

All primer sequences used in this study are listed in Table S3. 

## 4. Conclusions

We find that expressions of two sucrose metabolic genes *HpINV2* and *HpSuSy1* are correlated well with the elevated accumulation of glucose and fructose during pitaya fruit maturation. One nuclear-localized transcriptional activator HpWRKY3 is identified. More importantly, HpWRKY3 directly bind to *HpINV2* and *HpSuSy1* promoters, subsequently activating their transcriptions. Our findings shed novel light on the transcriptional regulatory mechanisms underlying sugar accumulation during fruit maturation, thereby laying the foundation for exploring new effective techniques to improve fruit quality.

## Supplementary Materials

Supplementary materials can be found at https://www.mdpi.com/1422-0067/20/8/1890/s1.
