# Supplementary material for: Pitaya HpWRKY3 Is Associated with Fruit Sugar Accumulation by Transcriptionally Modulating Sucrose Metabolic Genes HpINV2 and HpSuSy1
Source: Int J Mol Sci. 2019 Apr 17;20(8):1890. doi: 10.3390/ijms20081890 (PMC6514986; doi:10.3390/ijms20081890)
Supplement: Supplementary file 1 [file ijms-20-01890-s001.pdf]

Supplementary materials

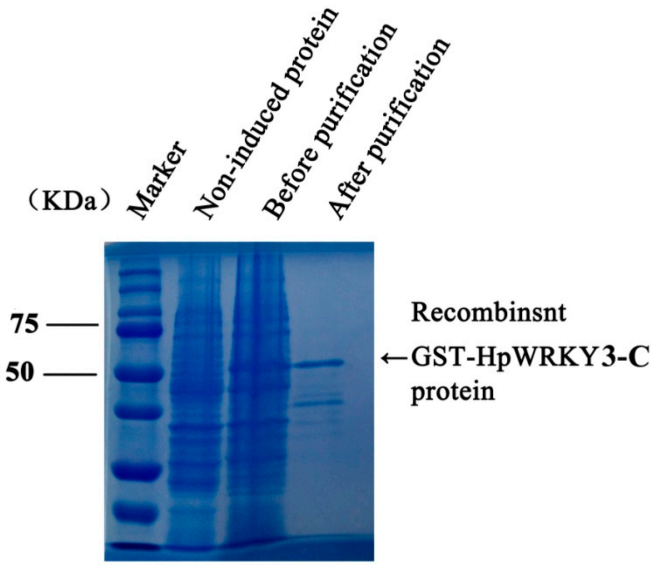

Figure S1. SDS-PAGE gel stained with Coomassie blue demonstrating affinity purification of the recombinant GST-WRKY3-C terminal.

Table S1 Main regulatory motifs found within the *HpINV2* promoter

Factor or site name	Signal sequence	Site	Function
AE-box	AGAAACTT	197(+)	part of a module for light response
Box 4	ATTAAT	720(+)	part of a conserved DNA module involved in light responsiveness
CAAT-box	CAAT	28(+),42(+),392(-),694(+),730(-),777(+),811(-), 1091(+),1099(-),1164(+)	common cis-acting element in promoter and enhancer regions
	CAAAT	163(+),391(-),399(-),470(+),668(-),1080(-), 1088(-),1208(-)	
	CCAAT	1073(+), 1098(-)	
GC-motif	CCCCCG	115(+)	enhancer-like element involved in anoxic specific inducibility
GCN4_motif	TGAGTCA	1004(-)	cis-regulatory element involved in endosperm expression
Myb-binding site	CAACAG	1011(-)	MYB binding site
MYC	CATTTG	1086(+),1208(+)	MYC recognition elements
TATA-box	TACAAAA	191(-),366(-),445(-)	core promoter element around -30 of transcription start
	TATA	293(-),303(-),330(-),341(-),375(-),399(-),409(-), 439(-),456(-),471(-),496(-),504(-),601(-),634(+), 690(+),741(+),795(+),800(+),831(+),933(+)	
	TATTTAAA	101(+),559(-)	
TCT-motif	TCTTAC	312(-),856(-)	part of a light responsive element
W box	TTGACC	1123(-)	WRKY binding site
	TTGACT	581(+)	

Table S2 Main regulatory motifs found within the *HpSuSy1* promoter

Factor or site name	Signal sequence	Site	Function
ARE	AAACCA	186(-),809(+)	cis-acting regulatory element essential for the anaerobic induction
		809(+)	
ATC-motif	AGTAATCT	1095(-)	part of a conserved DNA module involved in light responsiveness
CAAT-box	CAAT	68(-),72(-),206(+),424(+),494(-),921(-),1081(+),1151(-), 1174(+),1267(-),1269(+),1280(-),1374(-),1404(-),1415(-), 1468(-),1470(+),1562(-)	common cis-acting element in promoter and enhancer regions
	CAAAT	160(-),263(+),517(+),601(-),643(-),673(+),678(+),734(+), 775(+),788(+),1263(+),1360(-),1390(-)	
CAT-box	GCCACT	153(+),1305(-)	cis-acting regulatory element related to meristem expression
CGTCA-motif	CGTCA	694(-)	cis-acting regulatory element involved in the MeJA-responsiveness
ERE	ATTTCATA	1231(-)	Ethylene-responsive element
GATA-motif	AAGGATAAGG	703(-),1578(+)	part of a light responsive element
GC-motif	CCCCCG	34(+)	enhancer-like element involved in anoxic specific inducibility
GT1-motif	GGTTAA	394(+)	light responsive element
MBS	CAACTG	1488(-)	MYB binding site involved in drought-inducibility
MYB	CAACCA	847(+)	MYB binding site
MYC	CATTTG	161(+),734(-),788(-),1263(-),1269(+),1470(+)	MYC recognition elements
TATA-box	TACAAAA	512(+),813(+),905(+),1479(+)	core promoter element around -30 of transcription start
	TATA	89(-),279(-),332(-),352(-),504(-),531(-),542(-),577(-), 595(-),994(+),1028(+),1213(+),1440(+),1515(+),1594(+)	
TCA-element	CCATCTTTTT	762(-)	cis-acting element involved in salicylic acid responsiveness
TCT-motif	TCTTAC	365(-)	part of a light responsive element
TGA-element	AACGAC	940(+)	auxin-responsive element
W box	TTGACC	1414(+)	WRKY binding site
	TTGACT	1485(+),1403(+)	
WUN-motif	AAATTTCCT	1194(+)	wound-responsive element

Table S3 List of primers used in this study.

Assay	Primer sequence	Restriction Site
**RT-qPCR**	*HpINV1-qPCR-F:* TCAATCCTCGCCATCGAACTGC	
	HpINV1-qPCR-R: GGACCACGCCCCATTTTTGTCA	
	HpINV2-qPCR-F: ATGGTCCACTGTACCACAAGGG	
	HpINV2-qPCR-R: GCCATCTGGCAAGATCGTAGC	
	HpSuSy1-qPCR-F: AGGTATGTGGCTCAGGGGAAAG	
	HpSuSy1-qPCR-R: GCTATGGCAACGAAGGGAGG	
	HpSuSy2-qPCR-F: ATAGGCAATGGGGTTGAGTTCC	
	HpSuSy2-qPCR-R: TCAAGAGCCCTCAGGTTCTGAA	
	HpWRKY3-qPCR-F: CCTTTATCTCTGAGTCAACGCC	
	HpWRKY3-qPCR-R: ACAATCATGGAAGAAGGCTGTG	
**Promoter and Gene isolation**	*HpINV2 PRO-SP1:* TTGCAGCATATCATTTGTCCAAGCAAACTC	
	HpINV2 PRO-SP2: CCGCCGGAGACTCCCTGCCGTCTAGGATGA	
	HpINV2 PRO-SP3: GTTGGGCTGGGTCCGATNNNNNNNNNCCGTGG	
	HpSuSy1 PRO-SP1: CTATGGCAACGAAGGGAGGGACGACGATTG	
	HpSuSy1 PRO-SP2: TCATCGCCGATCACCTTTTCAAGTTCATCAAT	
	HpSuSy1 PRO-SP3: ACGATGAGCAGAGAGAGNNNNNNNNNCTTTCT	
	HpINV2 PRO-F: AGTTGTTTGGAGTTGGAGAGGG	
	HpINV2 PRO-R: CATTGAGGAAGAGAAGAGTTTTGTGT	
	HpSuSy1 PRO-F: ATATAACTTTGTCTGAGAAGGAAAAGGG	
	HpSuSy1 PRO-R: CATTGGAGATGGACGGTTCTAGA	
	HpWRKY3-F: ATGCCGGAAAACGATGCCGTG	
	HpWRKY3-R: CTATGTTATTCTCTCTTGTTTTAAATGGAG	
**Yeast One Hybrid**	*HpINV2 PRO-pAbAi-F: AATTGAAAAGCTTGAATTCGAGCTC*CCTTCTGCTCAACGAATTTTACAAGGTCAA	***Sac* I**
	*HpINV2 PRO-pAbAi-R: ACAGAGCACATGCCTCGAGGTCGAC*AATTTATTTAAAGAGTTGTATTCGATTT	***Sal* I**
	*HpSuSy1 PRO-pAbAi-F: AATTGAAAAGCTTGAATTCGAGCTC*AATATAACTAATGATAAATAGAAAACTATC	***Sac* I**
	*HpSuSy1 PRO-pAbAi-R: ACAGAGCACATGCCTCGAGGTCGAC*CAATGGCAAGAAAAAGTGTCGTTAGAGGTT	***Sal* I**
	*HpWRKY3-pGADT7-F: GGAGGCCAGTGAATTC*ATGCCGGAAAACGATGCCGTG	***EcoR* I**
	*HpWRKY3-pGADT7-R: CGAGCTCGATGGATCC*CTATGTTATTCTCTCTTGTTTTAAATGGAG	***Bam*H I**
**EMSA**	*HpWRKY3-C-pGEX-F: GGTTCCGCGTGGATCC*ATGCCAGCTGATGATGGCTATAATTGGAGG	***Bam*H I**
	*HpWRKY3-C-pGEX-R: AGTCACGATGCGGCCGC*TGTTATTCTCTCTTGTTTTAAATGGAG	***Not* I**
	HpINV2 PRO-Probe-F: ACAAGGTCAAACCCTAAAATTTTAATTGGGGGCAATTTGGTCATTTG	
	HpINV2 PRO-Probe-R: CAAATGACCAAATTGCCCCCAATTAAAATTTTAGGGTTTGACCTTGT	
	HpINV2 PRO-Probe-Mutant-F: ACAAAAAAAAAACCCTAAAATTTTAATTGGGGGCAATTTGGTCATTTG	
	HpINV2 PRO-Probe-Mutant-R: CAAATGACCAAATTGCCCCCAATTAAAATTTTAGGGTTTTTTTTTTGT	
	HpSuSy1 PRO-Probe-F: GCACTGTGTTTATGATAATTGACCCGTTATTGACTGCCAAAAATTTGAATTTT	
	HpSuSy1 PRO-Probe-R: AAAATTCAAATTTTTGGCAGTCAATAACGGGTCAATTATCATAAACACAGTGC	
	HpSuSy1 PR -Probe-Mutant-F: GCACTGTGTTTATGATAAAAAAAAACGTTAAAAAAAAGCCAAAAATTTGAATTTT	
	HpSuSy1 PRO-Probe-Mutant-R: AAAATTCAAATTTTTGGCTTTTTTTAACGTTTTTTTTATCATAAACACAGTGC	
**Subcellular localization**	*HpWRKY3-pEAQGFP-F: CAAATTCGCGACCGGT*ATGCCGGAAAACGATGCCGTG	***Age* I**
	*HpWRKY3-pEAQGFP-R: TGCTAGTCATACCGGT*TGTTATTCTCTCTTGTTTTAAAT	***Age* I**
**Trans-activation Activity Assay**	*HpWRKY3-pGBKT7-F: GCCATGGAGGCCGAATTC*ATGCCGGAAAACGATGCCGTG	***EcoR* I**
	*HpWRKY3-pGBKT7-R: GGCCGCTGCAGGTCGACG*CTATGTTATTCTCTCTTGTTTTAAATGGAG	***Sal* I**
	HpWRKY3-pBD-F: *TCGCCGACCGGTAGGCCT*ATGCCGGAAAACGATGCCGTG	***Stu* I**
	HpWRKY3-pBD-R: *AACCAGAGTTAAAGGCCT*CTATGTTATTCTCTCTTGTTTTAAATGGAG	***Stu* I**
**Transient expression**	*HpINV2 PRO-LUCReporter*-F: *TATAGGGCGAATTGGGTACC*AGTTGTTTGGAGTTGGAGAGGG	***Kpn* I**
	HpINV2 PRO-LUCReporter-R: *TTGGCGTCTTCCATGG*TGAGGAAGAGAAGAGTTTTGTGT	***Nco* I**
	HpSuSy1 PRO-LUCReporter-F: *TATAGGGCGAATTGGGTACC*ATATAACTTTGTCTGAGAAGGAAAAGGG	***Kpn* I**
	HpSuSy1 PRO-LUCReporter-R: *TTGGCGTCTTCCATGG*TGGAGATGGACGGTTCTAGAGAAC	***Nco* I**
	*HpWRKY3-pEAQ-F: CAAATTCGCGACCGGT*ATGCCGGAAAACGATGCCGTG	***Age* I**
	*HpWRKY3-pEAQ-R: AGTTAAAGGCCTCGAG*CTATGTTATTCTCTCTTGTTTTAAATGGAG	***Xho* I**

Text 1. Nucleotide sequences of *HpINV2* and *HpSuSy1* promoters. W-box motif were underlined. The probe nucleotide sequence used in EMSA assay is indicated in bold. Translation start site (ATG) was shown in yellow.

>*HpINV2*
AGTTGTTTGGAGTTGGAGAGGGTTCTTGAGGTTGAAAGAGAGAGAGCATTTGTTGAGAAAGA
ATGAGTCCAAACAGTAAACCTCAAAGTAGGCAATTCTACTTAAATATTCTTCCTTCTGCTCAAC
GAATTTT**ACAAGGTCAAACCCTAAAATTTTAATTGGGGGCAATTTGGTCATTTG**CCTTTCTCC
AATAATTTTTCCCACTTACTTTTTCTCCAAAAGCAACACCAAAATGAATTAGTTGCATACCTGTT
GATGACTCACTTACCAGTAAATTTTCTTAAATTAAGGGTCTGTTCTATTTCGCGTTTTAGATAGAT
AGATAGATATAAGGAGATGTAAGGAACTTTAAGAAGCTGTAAAGAGATGTAAGGAGATGTAAG
GAGCTGTAAGGAAATGTAAGAAGATGTAAGGAGCTGTAAGGATAAATATAAGGAGTTGTTGAA
TTGTAGATGAGTTGTATAGTATAATTTATTTTACAATGAACGTTTTGGATGAAAAATAATAATATG
AAAATAATATACTTATTGAAAATAAATAATTAATTAAGTTAATTACACAACAATAAAATATAATAA
TCCCCATAAATTTGGTGAATAAGATTTCGTCCACTTGAGGTGATTTTTTATATGAACATTAGTTTG
ATCGAATAAGCAAAGTATAATAAAATTTTTTTTTTTGACTAAATCGAATACAACTCTTTAAATA
AATTCCTATTTTCAGTAAATAATAATAAAAATAAATGTATGCTCATCTATAATAATATATATTTTATG
TCGAAACTCAAATATAATTTACGGCGTTATATTATGTTTTTATGTATATGTAAATGTCTTGTTCATA
AGATATTTATAAATTTGTATATTGGTAGTATCATTAAAAAATATACATATTTTTGTAACTAAAATAA
ATAAATGCTATAGAAAATCTATAAGATGTAAGGAGATGTAAGAAAATATAATAAAATATAAAAAA
CTGTAAAAAAATGTAAACTCTTAACTAAATTCTAAAAAAATGCAAAATAGAACCGGAGCTAGA
CACTTTTCTTTAAAAAAAAGAGAGAGAAACTTTTGTACACACTCGTGCATCTACCAGCCAAAT
CCAGAGTTGCCACCTCCCACTAACCCCAAGCCCGAGCACCCCCCCCCCGCGCCTCTCTATTTAA
ACCCATTCTTTCTCCTCCCTCCTCCCTCCTCCCTCCTCCCTCCATTCTCAATCTCAACCTCAACC
TCAATCAACACAAAACTCTTCTCTTCCTCAATG

>*HpSuSy1*
ATATAACTTTGTCTGAGAAGGAAAAGGGTACGCATTGTTAGAACTAGTTATGATCGATGATGTG
GACGCCCTAAAACTAATATAACTAATGATAAATAGAAAACTATCAGTTGACTTACAAAATGCAAT
TGTTGTGATGTTGAAATATTAAAGGTTATAAAAA**GCACTGTGTTTATGATAATTGACCCGTTAT**
TGACTGCCAAAAATTTGAATTTTGGGTTATTGGATGAGAATAATTTGAGCTTTAAAAATAGATT
TCAGTGTTTCGAACTTTGCGGATATTTATTTTCAGTGGCCAATATCATTTTAGATCGTATTGTTTT
AGGCAATTGCAAATGATATTACCCGTAAAGCAACACAAAGATATGAAATTAAAGTTACCTATAC
TCATTTTGAAGTTTAAATTTCCTTAAAAAAAATACAATCTAAAATGATATTTAGACTATTGAAAA
AATAAATACCGCAAAAGTTCAAGATGCTAAAAGTTATTTTTAAATTTTAGATTACTCCCGTTCAA
TACCTTCAAAGTTCAGATTCTGCACATCCGATTATCCTAATTAAACCTACTATAATTTTTTTTAGA
ATATCTCGCGATCATAATTATATAATAAAATAAAGCATCTTCATAAATAATAATTATTCATAACTAA
CCTCTAACGACACTTTTTCTTGCCATTGTAGAATGCGATGTACAAAATAGTTTCCTTCCCATAAT
CAAAGGCAAGAAAAGCAAACCGTCCCCCCCCCCCAACCACAAAAAAATAAAAAATCGGGGAC
AAAAGTACAAAACCATGAAATTAACGGCGACAAATGTACCCATCAAATCAAACTTGAAAAAGA
TAGCCTTTGAATCTAATTAGCCAAATGGAAAATGCGTACTATGACACTAATACATTATCCTTGAC
GTATCAGACTTGCAAATCAAATTTCTCCCTACTAACAAAGGAAGAGAATTTGATACTAAAGCCA
AACTGTTTATTTTTAAGTTTTGAAAATTTGATATATTAATTTTTATGATTATAATTTTTAAATTTACT

TTTAAGCATCATTACTTATATATTTTTTATAATTTAGTTCTCAAATTACAAAAGTATATTTATTATTG
TCATTTTTGTTTTTTTCTTTAAAACAAAAACTCTAAAAATCATCTTTTCGTTATTAGATAATTTATC
AATAATGTTGTGGGTACTTAAAAAAGAGAGGTTAATTTTTTTTTAAACATGAATAGTAGTAAGAA
TATTTTTATAATTCTAAAGCTAAATTTATAAAAAATTCATAAATAAAGTATTTAATAATAAATTTAA
AAATATTAAAACTATAAAATTAATGTATCAAATTTTTGTGGTAACTAAAAGTTAATAGTTATGCCA
AAACCCACACGACGCTTTTCAATGTGCTCATATTCACTGTGGTTTCCCAGCGGCAAGGCCCCCC
CATTTGGTGCCACTTGTTCTGGTGGTATTTTACCACCACTCACCTTTCGCAGTTCCAATTTTCTC
TCTCCTTTATATACCCCCTCTCTTATTGATTGTTCTTCGCTTGGCTCTACTACTTTCTTCTCCCCCC
GACCTCGTTCTCTAGAACCGTCCATCTCCAATG

Text 2. Sequence of HpWRKY3.

>*HpWRKY3*

ATGCCGGAAAACGATGCCGTGACTTCCACGAAACCGCCGCCGGCGCCGCGGATGGCGGTGGG
GCAGCTGCAGCCACCGACTATTACACTGCCGCCGCGTGGTGGGGTCGACTCCATTTTTTCTGGG
TCCGGGTTCGGAGCTAGCCCAGGCCCGATGACCCTGGTCTCCAGCTTCTTCTCTGAGAATGAC
CCGGATTCTGATTGTCGTTCTTTCTCTCAGCTCTTAGCTGGCGCCATGGCGTCTCCCCGACCGG
CTTCCGAGAACAGCTCTAGTGAACCGGACCATCTCCGGTTCAGGCAGAGCAGGCCGGCCGGT
CTGGTGGTTTCTCAGCAGCAAGGGGTTTTCACCATTCCGCCCGGGCTTAGTCCTGCTAGCTTGC
TCGATTCTCCTGGTTTATTCCCTGGTCAGGGGCCATTTGGGATGTCTCACCAGCAAGCGCTAGC
TCAAGTTACGGCTCAAGCTGTACAAGCACAAGCTAACTTGCATGTGCAAGGCAATTTTCCATCT
TCCCTCGGTATAGGTCTTCCGGGATCATCTCCATTTCCAGCCTTTATCTCTGAGTCAATGCCACA
AAATGTGACAAGTTCTGCCCTAGATTCTACCGAGATAAAAGAACCGGCACAGTACTCTGGCAT
GGACCAAAGATCACAGCCTTCTTCCATGATTGTTGATAAGCCAGCTGATGATGGCTATAATTGG
AGGAAATATGGGCAGAAGCAAGTTAAAGGGAGTGAATTTCCCCGAAGTTATTACAAATGTACA
CACATTAATTGCCCTGTCAAGAAGAAGGTGGAGCGTTCTCTTGATGGCCAAGTGACCGAGATA
ATCTATAAGGGCCAGCACAATCATCCGCCACCACAAAAACGTTCTAAAGAGAGCGGAAATTCT
AATGGAAACTTGGGAAGTCAAGGAAACCCTGAATTAAACATGAATAGCACGAATGAGGGCATG
ACTTACTCATTGTCTAGAAGGGATCAGGAATCTAGCCAAGCTACAGCAGAGCATTTGTCTAGTG
ATGAGGAGGAAGTGGGCAATGATGAAACTAGAGAATCTGAGGATGAACCTGATGCCAAGAGA
AGGAACACAGAACTCCGGGTACCAGAGCAAGCTGTTTCACATAGGACAGTCACTGAACCCAA
GATTATTGTTCAAACAACTAGTGAAGTTGATCTTTTGGACGATGGTTATAGGTGGCGCAAGTAT
GGACAAAAAGTTGTCAAAGGAAATTCATATCCGAGGAGCTATTACAAATGCACTCACCCAGGG
TGTAATGTTCGTAAGCATATTGAGCGGGCAGCCTCAGACCCTAAAGCAGTGATAACAACATACG
AGGGGAAGCACAGTCACGATGTTCCAGCTGCTAAGACGAGCAGCCATAACACATCCAACAGC
AGCTTTTCACAGCCAAGACCGCCTAGTTTGGCAGCGAATAATAACTCCTATGTTGGGAAGACG
GATTTACAAGTAATCAACAGCCTGTAGCCCTTCTCCATTTAAAACAAGAGAGAATAACATAG
